# Demographic change and HIV epidemic projections to 2050 for adolescents and young people aged 15-24

**DOI:** 10.1080/16549716.2019.1662685

**Published:** 2019-09-12

**Authors:** Aleya Khalifa, John Stover, Mary Mahy, Priscilla Idele, Tyler Porth, Chibwe Lwamba

**Affiliations:** a Division of Data, Research and Policy, United Nations Children’s Fund, New York, NY, USA; b Center for Modeling, Planning and Policy Analysis, Avenir Health, Avenir Health, Glastonbury, CT, USA; c Department of Strategic Information and Evaluation, Joint United Nations Programme for HIV/AIDS, Geneva, Switzerland

**Keywords:** HIV, HIV/AIDS, epidemiology, demography, epidemic modelling

## Abstract

**Background**: Ending AIDS as a public health threat by 2030 is a significant challenge, as new HIV infections among adolescents and young people have not decreased fast enough to curb the epidemic. The combination of slow HIV response and increasing youth populations 15–24 could affect progress towards 2030 goals.

**Objective**: This analysis aimed to describe global and regional trends from 2010–2050 in the HIV epidemic among adolescents and young people by accounting for demographic projections and recent trends in HIV interventions.

**Methods**: 148 national HIV estimates files were used to project the HIV epidemic to 2050. Numbers of people living with HIV and new HIV infections were projected by sex and five-year age group. Along with demographic data, projections were based on three key assumptions: future trends in HIV incidence, antiretroviral treatment coverage, and coverage of antiretrovirals for prevention of mother-to-child transmission. Results represent nine geographic regions.

**Results**: While the number of adolescents and young people is projected to increase by 10% from 2010–2050, those living with HIV is projected to decrease by 61%. In Eastern and Southern Africa, which hosts the largest HIV epidemic, new HIV infections among adolescents and young people are projected to decline by 84% from 2010–2050. In West and Central Africa, which hosts the second-largest HIV epidemic, new infections are projected to decline by 35%.

**Conclusions**: While adolescents and young people living with HIV are living longer and ageing into adulthood, if current trends continue, the number of new HIV infections is not projected to decline fast enough to end AIDS as a health threat in this age group. Regional variations suggest that while progress in Eastern and Southern Africa could reduce the size of the epidemic by 2050, other regions exhibit slower rates of decline among adolescents and young people.

## Background

The global community has committed to ending AIDS as a public health threat by 2030. This means the number of new HIV infections and AIDS-related deaths must decrease by 90 per cent between 2010 and 2030 [1]. However, this goal will not be achieved unless greater attention is dedicated to preventing HIV infection among adolescents and young people. In 2017, an estimated 3.9 million [2.1–5.7 million] adolescents and young people aged 15–24 were living with HIV. About 61 per cent of adolescents and young people living with HIV are adolescent girls and young women (AGYW), and about 78 per cent live in sub-Saharan Africa. While new HIV infections decreased by 20 per cent among adolescents and young people between 2010 and 2017, today they account for 36 per cent of new HIV infections among adults aged 15 and above. About 1,600 adolescents and young people become infected with HIV every day [2].

To end AIDS by 2030, the United Nations Joint Programme on HIV/AIDS (UNAIDS) developed the Fast-Track agenda. Under this agenda, the 95 – 95 – 95 goals for 2030 specify that 95 per cent of people living with HIV should know their HIV status, 95 per cent of those who know their status should be on antiretroviral treatment, and 95 per cent of those on treatment should be virally suppressed and sustained. The strategy also calls for a reduction of the current 1.6 million [1.3–2.1 million] annual number of new HIV infections among adults to 200,000 new HIV infections among adults by 2030 [1,2]. The Super – Fast Track agenda was set for 2020 to accelerate progress towards these 2030 goals for child, adolescent and young populations. Specifically, it calls for a reduction in the annual number of new HIV infections among adolescent girls and young women to 100,000 in 2020 [3]. However, in 2017 alone there were 340,000 [200,000–490,000] new HIV infections among adolescent girls and young women [2]. This means that new HIV infections among this population have been decreasing at an average annual rate of 3 per cent between 2010 and 2017, while a 13 per cent average annual rate of decrease has been required to achieve less than 100,000 new infections by 2020. It is clear from current estimates that the HIV response is off track for this 2020 goal.

HIV prevention has been particularly challenging in this population due to issues with social norms, social vulnerability, high-risk sexual behaviour, policy barriers, poor care-seeking behaviours and access to services [4–7]. HIV testing coverage remains low in this age group for these same reasons. In South Africa, the country with the highest burden of HIV in the world, only 38 per cent of adolescent girls and 29 per cent of adolescent boys in the general population report testing for HIV in the last 12 months and receiving the results of the test [8]. Even among those living with HIV in the United States, only an estimated 41 per cent of HIV-positive young people aged 13–29 know their HIV status [9]. Adolescents and young people living with HIV also exhibit low adherence to antiretroviral therapy (ART). For example, a meta-analysis from 53 countries found that 62 [57–68] per cent of adolescents and young people living with HIV aged 12–24 adhered to therapy [10]. This is of concern because 90 – 90 – 90 goals for 2020 call for 73 per cent prevalence viral load suppression among people living with HIV, which cannot be achieved without adequate adherence to ART. The Namibia Population-based HIV Impact Assessment (PHIA) found that 82 per cent and 70 per cent of adult women and men living with HIV, respectively, were virally suppressed, but only 65 per cent of adolescent girls and young women and 61 per cent of adolescent boys and young men were virally suppressed [11]. Evidence shows that the HIV response is off-track for global targets among adolescents and young people. To address this problem, more evidence is needed to monitor progress towards global HIV goals, understand barriers in HIV prevention, care and treatment, and improve interventions for this age group.

Demographic shifts could impact the HIV response and pose an additional complication in preventing HIV infection and improving treatment among adolescents and young people in countries experiencing population growth in this age group. Age structures have changed over time and are projected to continue changing as countries undergo demographic transition. The recent 2017 Revision of World Population Prospects shows that while fertility rates are on the decline globally, some parts of the world are still projected to face population growth in adolescent and youth age groups between now and 2050 [12]. This projected growth is largest in the region most affected by HIV: sub – Saharan Africa. Population change may also affect the absolute number of new HIV infections and total number of people living with HIV in parts of the world where HIV incidence has increased or remained the same since 2010, namely Latin America and the Caribbean, East Asia and the Pacific and Eastern Europe and Central Asia.

By 2050, the population aged 15–24 is expected to increase by 10 per cent globally. This is mostly driven by sub-Saharan Africa, where the population aged 15–24 is projected to more than double [12]. Sub – Saharan Africa is also home to 72 per cent of new HIV infections among adolescents and young people, and the number of new HIV infections among adolescents and young people in the region has only decreased by 22 per cent since 2010.

The combination of a growing population of young people, high fertility rates and persistent HIV incidence could impact the rate of reduction of new HIV infections in various geographies. This paper uses an HIV epidemic model to assess the influence of HIV programme response and demographic factors such as trends in population size of adolescents and youth, fertility rate, and HIV incidence on the future of the HIV epidemic for adolescents and young people from 2010 to 2050. The ultimate objective of this analysis is to evaluate whether the HIV response is on track for global goals to end AIDS among adolescents and young people by 2030.

## Methods

HIV projections were generated for 148 countries from the most recent country-produced HIV estimates using the AIDS Impact Model (AIM) in Spectrum software (Avenir Health, Glastonbury, CT, USA). 21 countries with a 2018 Spectrum file and no historical HIV incidence data available (mostly in the Middle East, North Africa and Western Europe) were excluded from the analysis. Countries may not have historical HIV incidence data if no population-based survey has been conducted, or no data are available from routine surveillance or HIV programme data. The remaining countries have no Spectrum file at all. The Spectrum model utilizes both historical and latest demographic, epidemiologic and HIV programme data to inform HIV estimates and measure progress in the epidemic response [13,14]. Demographic data are gathered from the United Nations Population Division’s World Population Prospects or national census data belonging to that country [12]. Epidemiologic data include scientifically-informed parameters and prevalence, incidence, or mortality data from surveillance, surveys and special studies [15–18]. Finally, HIV programme data are imported from national health information systems. Methods are documented in the UNAIDS *Annex on Methods* [19].

In the country-produced files, HIV incidence and prevalence estimates are projected five years into the future. However, for this analysis the timeframe in the model was extended to 2050 in order to assess possible demographic trends, which cannot be adequately assessed using five years of projected estimates. This required various assumptions about how to project HIV incidence, and coverage of antiretroviral therapy (ART) and prevention of mother-to-child transmission (PMTCT) services past 2017 (the last year in the model with observed data).

Trends from 2013–2017 of ART and PMTCT coverage were extrapolated into the future using a log-linear curve to reflect typical coverage trajectories in these key HIV interventions. Coverage rates were held constant once they reached 95 per cent. For most countries, HIV incidence trends from 2017–2022 were projected until 2050 using a log-linear curve. A linear projection was applied for all countries where incidence was increasing from 2017–2022.

Two outputs were extracted from the Spectrum AIDS impact model (AIM) to assess the trajectory of the HIV epidemic over time and by five-year age group and sex for each country by number of new HIV infections and people living with HIV. Although the assumptions and projections were calculated at the national level, these were aggregated from the regional and global level for this analysis, with specific focus on the 15–24 age group. All projections were compared to 2010, which was the base year for the *Political Declaration on HIV and AIDS: On the Fast Track to Accelerating the Fight against HIV and to Ending the AIDS Epidemic by 2030* [20]. Decade-long increments are used to compare HIV projections over time: 2010, 2020, 2030, 2040 and 2050. All results were rounded to the nearest thousand to avoid false precision.

Nine geographic regions based on UNICEF classification were used in this analysis: East Asia and the Pacific, Eastern and Southern Africa, Eastern Europe and Central Asia, Latin America and the Caribbean, the Middle East and North Africa, North America, South Asia, West and Central Africa, and Western Europe.

## Results

The 148 countries included in this analysis represented about 95 per cent of adults aged 15 and above and nearly 100 per cent of adults aged 15 and above living with HIV in 2017. For adolescents and young people aged 15–24, the countries included in this analysis represented 97 per cent and 100 per cent, respectively, of the absolute population size and those living with HIV. The following results are organized by the two output indicators: number of people living with HIV and number of new HIV infections.

### Projected number of people living with HIV

**Figure 1.**
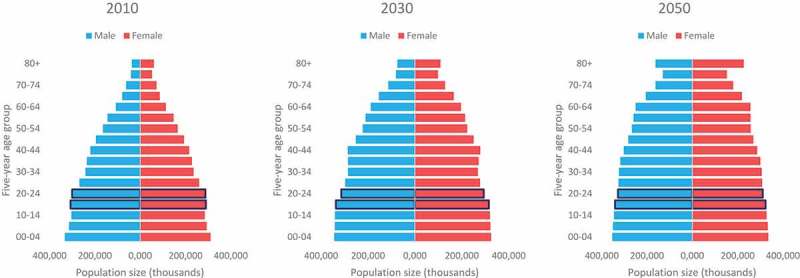
Population size (thousands) by age and sex, 2010, 2030 and 2050, UNAIDS 2018 estimates.

**Figure 2.**
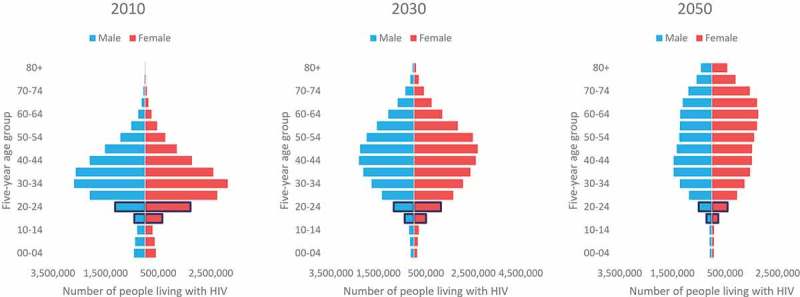
Number of people living with HIV by age and sex, 2010, 2030 and 2050, UNAIDS 2018 estimates.

The proportion of the population that is in the 15–24 age group is projected to decline from 18 per cent in 2010 to 14 per cent in 2050 (Figure 1). However, a more rapid pattern is projected for the population living with HIV from 2010 to 2050, as less adolescents living with HIV are projected to join that age group over time (Figure 2). In 2010, the majority of the global population living with HIV was under the age of 35, while in 2050 the majority of the population living with HIV is projected to be under age 55. Projections show a more dramatic ageing of the population living with HIV compared to the general population. By 2050, the majority of the general population is projected to be under age 40; suggesting that in 2050 the population living with HIV will be on average older than the general population. In 2010, there were about 3.9 million adolescents and young people aged 15–24 living with HIV (12 per cent of all people living with HIV). This number is projected to decline to 1.5 million (5 per cent of all people living with HIV) in 2050. Between 2010 and 2050, the number of adolescents and young people living with HIV is projected to decline by 61 per cent, compared to a 10 per cent decline in the general adolescent and youth population. By sex, the number of AGYW living with HIV are projected to decline by 65 per cent while the number of adolescent boys and young men (ABYM) living with HIV are projected to decline by 55 per cent.

The projected population living with HIV differs by region (Table 1). The total adult population aged 15–49 living with HIV is projected to decline by at least 50 per cent in Eastern and Southern Africa, East Asia and the Pacific and South Asia between 2010 and 2050. In these same regions, the population aged 15–24 living with HIV is projected to decline by at least 60 per cent.

**Table 1.** Number of people living with HIV by decade, region, age and sex, 2010–2050, UNAIDS 2018 estimates.

Region	2010		2020			2030			2040			2050		
	Estimate	% of adults aged 15–49	Estimate	% of adults aged 15–49	% Change since 2010	Estimate	% of adults aged 15–49	% Change since 2010	Estimate	% of adults aged 15–49	% Change since 2010	Estimate	% of adults aged 15–49	% Change since 2010
**Eastern and Southern Africa**														
Adults aged 15–49	14,476,000		15,841,000		9.4	12,284,000		−15.1	8,027,000		−44.6	5,218,000		−64.0
Adults aged 15–24	2,157,000	14.9	1,982,000	12.5	−8.1	1,459,000	11.9	−32.4	885,000	11.0	−59.0	548,000	10.5	−74.6
Girls and women aged 15–24	1,483,000	10.2	1,290,000	8.1	−13.0	913,000	7.4	−38.4	548,000	6.8	−63.0	336,000	6.4	−77.4
Boys and men aged 15–24	675,000	4.7	692,000	4.4	2.6	546,000	4.4	−19.0	337,000	4.2	−50.0	212,000	4.1	−68.5
**East Asia and the Pacific**														
Adults aged 15–49	2,129,000		2,099,000		−1.4	1,693,000		−20.5	1,279,000		−40.0	1,062,000		−50.1
Adults aged 15–24	300,000	14.1	205,000	9.8	−31.7	167,000	9.9	−44.3	135,000	10.5	−55.1	110,000	10.3	−63.4
Girls and women aged 15–24	123,000	5.8	76,000	3.6	−38.7	57,000	3.4	−54.0	44,000	3.5	−64.1	34,000	3.2	−72.3
Boys and men aged 15–24	177,000	8.3	129,000	6.2	−26.8	110,000	6.5	−37.6	91,000	7.1	−48.8	76,000	7.1	−57.3
**Eastern Europe and Central Asia**														
Adults aged 15–49	680,000		1,158,000		70.3	1,263,000		85.8	1,178,000		73.3	1,171,000		72.3
Adults aged 15–24	69,000	10.1	68,000	5.9	−1.5	88,000	7.0	27.5	92,000	7.8	33.2	72,000	6.1	4.2
Girls and women aged 15–24	33,000	4.8	34,000	2.9	2.5	44,000	3.5	34.9	45,000	3.8	35.9	35,000	3.0	7.4
Boys and men aged 15–24	36,000	5.3	34,000	3.0	−5.2	44,000	3.5	20.7	47,000	4.0	30.7	37,000	3.1	1.4
**Latin America and the Caribbean**														
Adults aged 15–49	1,386,000		1,662,000		20.0	1,633,000		17.8	1,386,000		0.1	1,168,000		−15.7
Adults aged 15–24	224,000	16.2	214,000	12.9	−4.5	187,000	11.4	−16.8	167,000	12.1	−25.4	142,000	12.2	−36.6
Girls and women aged 15–24	92,000	6.7	86,000	5.2	−6.6	76,000	4.7	−17.3	69,000	5.0	−25.6	58,000	5.0	−36.8
Boys and men aged 15–24	132,000	9.5	128,000	7.7	−3.0	110,000	6.8	−16.4	99,000	7.1	−25.3	84,000	7.2	−36.5
**Middle East and North Africa**														
Adults aged 15–49	119,000		150,000		26.3	156,000		31.1	145,000		21.5	141,000		18.8
Adults aged 15–24	16,000	13.7	14,000	9.1	−16.3	16,000	10.1	−4.0	16,000	11.0	−2.4	14,000	10.1	−13.1
Girls and women aged 15–24	7,000	5.6	6,000	4.1	−6.0	7,000	4.7	11.1	7,000	5.1	12.5	7,000	4.7	1.6
Boys and men aged 15–24	10,000	8.2	7,000	5.0	−23.3	8,000	5.4	−14.2	9,000	5.9	−12.5	8,000	5.3	−23.0
**North America**														
Adults aged 15–49	567,000		628,000		10.9	624,000		10.2	529,000		−6.7	432,000		−23.7
Adults aged 15–24	74,000	13.0	58,000	9.2	−21.4	48,000	7.7	−34.4	40,000	7.6	−45.3	33,000	7.7	−55.0
Girls and women aged 15–24	20,000	3.6	16,000	2.6	−19.7	14,000	2.2	−32.1	12,000	2.2	−43.5	10,000	2.2	−53.4
Boys and men aged 15–24	53,000	9.4	42,000	6.6	−22.1	35,000	5.5	−35.2	29,000	5.4	−46.0	24,000	5.5	−55.5
**South Asia**														
Adults aged 15–49	2,282,000		2,307,000		1.1	1,795,000		−21.3	1,363,000		−40.3	1,013,000		−55.6
Adults aged 15–24	260,000	11.4	231,000	10.0	−11.0	161,000	9.0	−38.0	103,000	7.6	−60.2	71,000	7.0	−72.8
Girls and women aged 15–24	121,000	5.3	108,000	4.7	−10.8	76,000	4.2	−37.6	48,000	3.6	−60.0	33,000	3.3	−72.6
Boys and men aged 15–24	139,000	6.1	123,000	5.3	−11.2	86,000	4.8	−38.4	55,000	4.0	−60.5	38,000	3.7	−73.0
**West and Central Africa**														
Adults aged 15–49	4,485,000		4,480,000		−0.1	4,389,000		−2.1	4,229,000		−5.7	4,094,000		−8.7
Adults aged 15–24	787,000	17.6	788,000	17.6	0.1	733,000	16.7	−6.9	620,000	14.7	−21.3	529,000	12.9	−32.9
Girls and women aged 15–24	488,000	10.9	474,000	10.6	−2.9	443,000	10.1	−9.2	375,000	8.9	−23.2	323,000	7.9	−33.9
Boys and men aged 15–24	299,000	6.7	314,000	7.0	5.0	290,000	6.6	−3.0	245,000	5.8	−18.1	206,000	5.0	−31.1
**Western Europe**														
Adults aged 15–49	313,000		311,000		−0.8	293,000		−6.6	268,000		−14.4	255,000		−18.6
Adults aged 15–24	37,000	11.7	31,000	10.1	−14.2	30,000	10.3	−17.3	28,000	10.4	−23.3	27,000	10.5	−26.7
Girls and women aged 15–24	13,000	4.3	11,000	3.7	−14.8	11,000	3.8	−17.6	10,000	3.8	−23.9	10,000	3.8	−26.9
Boys and men aged 15–24	23,000	7.4	20,000	6.4	−13.9	19,000	6.6	−17.2	18,000	6.7	−22.9	17,000	6.7	−26.5
**Global**														
Adults aged 15–49	26,437,000		28,635,000		8.3	24,130,000		−8.7	18,404,000		−30.4	14,555,000		−44.9
Adults aged 15–24	3,925,000	14.8	3,592,000	12.5	−8.5	2,890,000	12.0	−26.4	2,087,000	11.3	−46.8	1,545,000	10.6	−60.6
Girls and women aged 15–24	2,381,000	9.0	2,102,000	7.3	−11.7	1,641,000	6.8	−31.1	1,158,000	6.3	−51.4	845,000	5.8	−64.5
Boys and men aged 15–24	1,544,000	5.8	1,491,000	5.2	−3.5	1,249,000	5.2	−19.1	929,000	5.0	−39.8	700,000	4.8	−54.6

In West and Central Africa, the number of all adults living with HIV is projected to decrease by 9 per cent between 2010 and 2050, while the number of adolescents and young people living with HIV is projected to decrease by 33 per cent.

Further, the adult population aged 15–49 living with HIV is projected to increase in Eastern Europe and Central Asia and Middle East and North Africa between 2010 and 2050.

In Eastern Europe and Central Asia, the population aged 15–24 living with HIV is projected to increase by 28 per cent by 2030, but then growth is expected to taper and then decline so that the overall increase between 2010 and 2050 will only be 4 per cent. This is the only region with a projected increase in adolescent and youth populations living with HIV from 2010 to 2050.

### Projected number of new HIV infections

**Figure 3.**
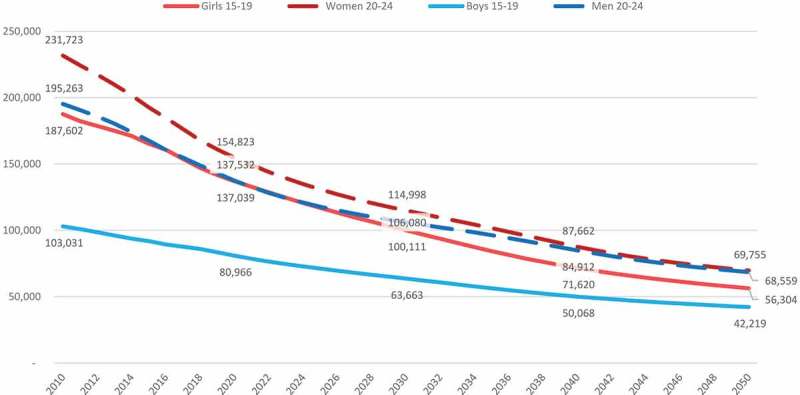
Number of new HIV infections among adolescent and young people, by sex and five-year age group, 2010–2050, UNAIDS 2018 estimates.

Projected numbers of new HIV infections also differ by age, sex and region. Globally, new HIV infections are projected to decline between 2010 and 2050, the most for adolescent girls aged 15–19 (70 per cent decline) and young women aged 20–24 (70 per cent decline) (Figure 3). The number of new HIV infections among ABYM is projected to decline by 59 per cent and 65 per cent, respectively. While adolescent boys only accounted for 14 per cent of new HIV infections among adolescents and young people in 2010, this is projected to account for 18 per cent in 2050. For all age groups, steeper reductions in the number of new HIV infections are projected to occur between 2010 and 2030 (3.1 average annual rate of reduction) compared to 2030 to 2050 (2.4 average annual rate of reduction).

By region, the largest reduction in new HIV infections among adolescents and young people from 2010 to 2050 is projected for Eastern and Southern Africa (84 per cent) (Table 2). In East Asia and the Pacific, North America, and South Asia, the number of new HIV infections among adolescents and young people is projected to decline by at least 57 per cent from 2010 to 2050. In Eastern Europe and Central Asia, new HIV infections among those aged 15–24 are projected to increase until 2040 and will decrease thereafter. In West and Central Africa, the region with the second – highest burden, the number of new HIV infections among adolescents and young people is projected to decrease by 35 per cent between 2010 and 2050 assuming incidence patterns follow current trends.

**Table 2.** Number of new HIV infections by decade, region, age and sex, 2010–2050, UNAIDS 2018 estimates.

Region	2010		2020			2030			2040			2050		
	Estimate	% of adults aged 15–49	Estimate	% of adults aged 15–49	% Change since 2010	Estimate	% of adults aged 15–49	% Change since 2010	Estimate	% of adults aged 15–49	% Change since 2010	Estimate	% of adults aged 15–49	% Change since 2010
**Eastern and Southern Africa**														
Adults aged 15–49	951,000		612,000		−35.7	400,000		−58.0	264,000		−72.2	181,000		−81.0
Adults aged 15–24	398,000	41.9	247,000	40.4	−37.9	154,000	38.6	−61.2	96,000	36.2	−76.0	62,000	34.5	−84.3
Girls and women aged 15–24	266,000	28.0	166,000	27.2	−37.5	104,000	26.0	−61.0	64,000	24.2	−76.0	41,000	22.6	−84.6
Boys and men aged 15–24	132,000	13.9	81,000	13.2	−38.7	50,000	12.6	−61.8	32,000	12.1	−75.8	22,000	11.9	−83.7
**East Asia and the Pacific**														
Adults aged 15–49	165,000		133,000		−19.4	105,000		−36.1	90,000		−45.3	82,000		−50.4
Adults aged 15–24	64,000	38.5	43,000	32.4	−32.2	32,000	30.6	−49.2	27,000	29.4	−58.3	23,000	27.8	−64.2
Girls and women aged 15–24	25,000	15.2	15,000	11.0	−41.9	10,000	9.5	−60.2	8,000	8.7	−68.7	7,000	8.0	−73.8
Boys and men aged 15–24	38,000	23.3	28,000	21.4	−25.9	22,000	21.1	−42.1	19,000	20.7	−51.5	16,000	19.8	−57.9
**Eastern Europe and Central Asia**														
Adults aged 15–49	97,000		123,000		27.0	118,000		20.9	106,000		9.4	102,000		5.4
Adults aged 15–24	19,000	19.9	17,000	13.6	−12.9	22,000	18.6	13.5	21,000	20.2	11.2	17,000	16.5	−12.2
Girls and women aged 15–24	8,000	8.6	8,000	6.2	−8.3	10,000	8.5	19.5	9,000	8.8	11.2	8,000	7.4	−9.8
Boys and men aged 15–24	11,000	11.2	9,000	7.4	−16.4	12,000	10.1	8.9	12,000	11.4	11.2	9,000	9.2	−14.0
**Latin America and the Caribbean**														
Adults aged 15–49	105,000		104,000		−0.5	97,000		−7.3	88,000		−16.3	78,000		−25.6
Adults aged 15–24	41,000	38.8	38,000	35.9	−7.7	33,000	33.8	−19.0	30,000	33.7	−27.3	26,000	33.1	−36.4
Girls and women aged 15–24	15,000	14.7	14,000	13.6	−7.8	13,000	13.0	−18.3	11,000	13.0	−26.3	10,000	12.8	−35.2
Boys and men aged 15–24	25,000	24.1	23,000	22.3	−7.7	20,000	20.9	−19.5	18,000	20.7	−27.9	16,000	20.3	−37.2
**Middle East and North Africa**														
Adults aged 15–49	12,000		11,000		−3.4	11,000		−8.6	10,000		−15.7	9,000		−20.0
Adults aged 15–24	3,000	29.7	3,000	24.6	−19.9	3,000	29.0	−10.8	3,000	30.3	−13.9	3,000	29.2	−21.4
Girls and women aged 15–24	1,000	11.3	1,000	10.4	−11.3	1,000	12.5	1.3	1,000	13.1	−2.1	1,000	12.9	−8.7
Boys and men aged 15–24	2,000	18.4	2,000	14.2	−25.1	2,000	16.4	−18.2	2,000	17.1	−21.2	1,000	16.2	−29.2
**North America**														
Adults aged 15–49	43,000		34,000		−21.3	28,000		−34.4	23,000		−46.0	19,000		−55.5
Adults aged 15–24	14,000	33.0	11,000	31.3	−25.3	9,000	31.4	−37.6	7,000	31.9	−47.8	6,000	31.7	−57.3
Girls and women aged 15–24	4,000	8.5	3,000	8.3	−23.6	2,000	8.3	−35.8	2,000	8.5	−46.3	2,000	8.5	−55.9
Boys and men aged 15–24	11,000	24.5	8,000	23.0	−25.9	7,000	23.0	−38.2	5,000	23.5	−48.3	4,000	23.3	−57.7
**South Asia**														
Adults aged 15–49	111,000		87,000		−21.4	68,000		−38.5	56,000		−49.6	50,000		−55.4
Adults aged 15–24	44,000	39.8	31,000	35.0	−30.8	21,000	31.0	−52.1	15,000	27.3	−65.4	12,000	24.4	−72.6
Girls and women aged 15–24	20,000	17.6	13,000	15.2	−31.7	9,000	13.5	−52.7	7,000	11.8	−66.1	5,000	10.6	−73.2
Boys and men aged 15–24	25,000	22.2	17,000	19.8	−30.1	12,000	17.5	−51.6	9,000	15.5	−64.8	7,000	13.9	−72.2
**West and Central Africa**														
Adults aged 15–49	271,000		249,000		−8.0	233,000		−14.0	218,000		−19.4	210,000		−22.3
Adults aged 15–24	127,000	46.8	115,000	46.3	−9.1	104,000	44.7	−17.9	90,000	41.0	−29.4	82,000	39.1	−35.0
Girls and women aged 15–24	77,000	28.4	69,000	27.9	−9.8	64,000	27.3	−17.5	55,000	25.2	−28.4	51,000	24.2	−33.7
Boys and men aged 15–24	50,000	18.4	46,000	18.4	−8.0	41,000	17.4	−18.5	34,000	15.8	−30.8	31,000	14.9	−37.1
**Western Europe**														
Adults aged 15–49	22,000		20,000		−10.4	18,000		−19.0	17,000		−23.7	16,000		−26.3
Adults aged 15–24	7,000	32.3	6,000	32.7	−9.3	6,000	34.3	−13.9	6,000	34.2	−19.1	6,000	34.3	−21.8
Girls and women aged 15–24	2,000	11.0	2,000	11.1	−9.4	2,000	11.6	−14.4	2,000	11.6	−19.8	2,000	11.6	−22.1
Boys and men aged 15–24	5,000	21.3	4,000	21.6	−9.2	4,000	22.7	−13.7	4,000	22.7	−18.8	4,000	22.6	−21.7
**Global**														
Adults aged 15–49	1,777,000		1,374,000		−22.7	1,078,000		−39.3	872,000		−50.9	748,000		−57.9
Adults aged 15–24	718,000	40.4	510,000	37.1	−28.9	385,000	35.7	−46.4	294,000	33.7	−59.0	237,000	31.7	−67.0
Girls and women aged 15–24	419,000	23.6	292,000	21.2	−30.4	215,000	20.0	−48.7	159,000	18.3	−62.0	126,000	16.9	−69.9
Boys and men aged 15–24	298,000	16.8	218,000	15.9	−26.8	170,000	15.7	−43.1	135,000	15.5	−54.7	111,000	14.8	−62.9

**Figure 4.**
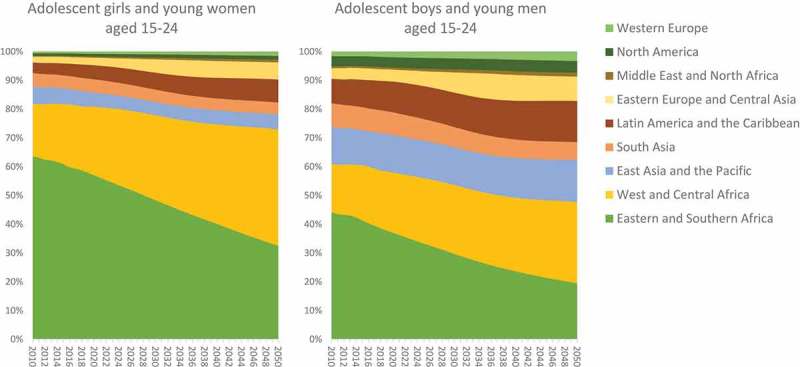
Percent distribution of new HIV infections among adolescents and young people by region, 2010–2050, UNAIDS 2018 estimates.

These regional projections result in sex-specific changes in the percent distribution of new HIV infections among adolescents and young people by region (Figure 4). Among AGYW aged 15–24, 64 per cent of new HIV infections occurred in Eastern and Southern Africa in 2010. By 2050, only 32 per cent of new HIV infections among AGYW are projected to occur in Eastern and Southern Africa – or a decrease from 270,000 in 2010 to 41,000 in 2050 in absolute numbers. By 2050, 40 per cent of new HIV infections among AGYW are projected to occur in West and Central Africa (compared to 18 per cent in 2010). Other regions that are projected to contribute more to the global total of new HIV infections among AGYW include Eastern Europe and Central Asia (2 per cent in 2010 to 6 per cent in 2050) and Latin America and the Caribbean (4 per cent in 2010 to 8 per cent in 2050).

Among ABYM aged 15–24, 31 per cent of new HIV infections occurred in Eastern and Southern Africa in 2010, and this is projected to decline to 17 per cent of new HIV infections by 2050, while 25 per cent is projected to occur in West and Central Africa (compared to 12 per cent in 2010). Other regions that are projected to contribute more to the global total of new HIV infections among ABYM include East Asia and the Pacific (9 per cent in 2010 to 13 per cent in 2050), Eastern Europe and Central Asia (3 per cent in 2010 to 7 per cent in 2050) and Latin America and the Caribbean (6 per cent in 2010 to 13 per cent in 2050).

## Discussion

These findings show that the total number of young people newly infected with HIV will not surge over time, given trends in population size, HIV incidence, and key HIV interventions. However, the pattern in HIV infections and age and sex structure of people living with HIV will differ by region given region-specific population dynamics and epidemic trends.

After 2010, the global youth bulge population from previous decades will finally age out of the 20–24 age group. However, the number of adolescents and young people aged 15–24 is projected to grow at a slow and steady rate until 2050. Unlike the general population, the age structure of the population living with HIV is projected to change dramatically over the next 30+ years. The proportion of all people living with HIV in the 15–24 age group is projected to decline as adolescents and young people age into adulthood. Since these projections do not mimic the general population age structure these global HIV projections are more a function of trends in the HIV response than of population change.

Findings on HIV projections differ by region. As progress is made in Eastern and Southern Africa, the relative burden of new HIV infections in adolescent and youth age groups is projected to tilt to other world regions.

The projections suggest that, with current trends, the 2020 Super – Fast Track targets are not likely to be achieved in any region. For example, this analysis projected about 292,000 new HIV infections among adolescent girls and young women in 2020 compared to the Super – Fast Track targets of less than 100,000 new HIV infections. Current trends in HIV incidence and intervention coverage must change if an AIDS Free Generation is to be achieved by 2030.While new HIV infections among adolescent girls and young women are projected to decrease at an average annual rate of −2 per cent between 2017 and 2030, the necessary rate of reduction should be −14 per cent for infections to decrease in this population in order to contribute to the global 2030 goal of under 200,000 new HIV infections among all people age 15 and above.

Finally, projections show that recent trends in the HIV response, independent of demographic change, may have a lasting effect on the future of the epidemic. The number of people living with HIV would be expected to change in the same proportion to the total population if population change were the only factor. Since projected numbers of people living with HIV are different from projected numbers of the general population, it is evident that demographic changes are not the only factor in HIV projections. Thus, this analysis suggests that improvements in the HIV response could change the trajectory of the HIV epidemic for the better, despite demographic factors. However, it would be helpful to quantify the contribution of demographic factors alone on HIV epidemic projections. For example, a recent UNAIDS analysis showed that if population growth had been stable, the number of new HIV infections among people in sub-Saharan Africa would have decreased by 19 per cent instead of 16 per cent between 2010 and 2016 [21].

### Limitations

This analysis includes some key limitations. First, some countries were excluded from the analysis. In 2018, 169 countries created a Spectrum file available through UNAIDS (representing 99 per cent of the global population). While some country models were excluded from this analysis due to a lack of historical HIV incidence data, the final set of 148 countries represent 97 per cent of the global adolescent and youth population and almost 100 per cent of the all adolescents and young people living with HIV. Second, projections are only as strong as the input data. Model inputs include population statistics, survey data and HIV programme data. The quality of HIV estimates depends on the robustness of input data, especially programme statistics. While the issue of data quality cannot be completely addressed, UNAIDS, WHO, UNICEF and other partners undertake a rigorous review of PMTCT and ART data to minimize some errors. The quality of HIV estimates also depends on the accuracy of inherent assumptions and algorithms in the model, of which scientific literature is reviewed biennially to implement any methodological changes. Knowledge of epidemic patterns and programme effects is constantly improving which can cause modelled estimates to change from one year to the next. The UNAIDS Reference Group recommends changes based on the latest scientific evidence but some gaps may remain [14,22–30]. For example, while the results suggest that HIV epidemic projections differ by sex in each region, information about incidence rate ratios outside of sub-Saharan Africa is relatively weak due to sparse surveillance and survey data, in addition to small sample sizes in both data types [31,32]. Thus, there is limited certainty around sex-specific projection patterns outside of sub-Saharan Africa.

Projections to 2050 assume that external factors will remain the same as they were in 2018. This model does not account for unforeseen changes in HIV treatment availability, HIV-related policies, or funding contexts.

Furthermore, this analysis aggregated country estimates to a regional level, thus masking country variations. It also used national HIV estimates by five-year age group and sex as the unit for analysis, which could mask sub-population HIV incidence trends. For example, a study of HIV case reports in South-eastern China found that the percent of new reported HIV cases in the 15–18 age group has decreased while the percent of new reported HIV cases in the 19–22 age group has increased from 2000 to 2015 [33]. Local trends and finer age groups may elucidate further contextual factors that play a role in the projected number of new HIV infections among adolescents and young people.

## Conclusion

The numbers of young people living with HIV are projected to decline globally if current trends in HIV infection rates, programmatic response, and population changes continue. However, HIV will remain a serious problem in regions where HIV testing, treatment and retention coverage remains low for this population group and where the adolescent and young adult population is expected to increase in the coming decades. Strong efforts are needed to ensure that the numbers continue to decline and to speed that decline to achieve global targets. Regions of the world with increasing HIV incidence like Eastern Europe and Central Asia must be targeted with locally appropriate interventions. HIV prevention must continue to be prioritized among adolescents and young people living in high – prevalent areas. In these areas, the contextual challenges to HIV prevention must be addressed [4]. Pre-exposure prophylaxis for adolescents at higher risk of HIV infection is one tool that can still be improved and brought to scale in high-prevalent areas, but more research is needed to inform effective implementation of this interventions in adolescent populations [34–36]. These HIV prevention challenges are often gendered. Adolescent boys and young men face different barriers to HIV prevention services compared to adolescent girls and young women [5,37]. These findings demonstrate that the end of the HIV epidemic is not close for adolescents and young people. By utilizing current trends in the HIV response in the epidemic model, these results illustrate which populations and regions may need more attention to end AIDS as a public health threat by 2030. While reducing HIV incidence in adolescence and young adulthood is critical to ending the epidemic, it will also be important to plan sustainable and integrated testing, care and treatment programmes for this age group- and as they age.

## Data Availability

The data that support the findings of this study are openly available in national HIV estimates files published through UNAIDS at http://www.unaids.org/en/dataanalysis/datatools/spectrum-epp.

## Paper context

HIV incidence among adolescents and young people is not decreasing fast enough to end AIDS as a public health threat by 2030. While experts fear that increasing youth populations could further impede progress, this paper demonstrates that improvements in the HIV response could change the trajectory of the HIV epidemic despite demographic factors. New HIV infections must decrease faster and HIV prevention programmes must appropriately target adolescents and young people to secure an AIDS-free generation.
